# Looking Through Paintings by Combining Hyper-Spectral Imaging and Pulse-Compression Thermography

**DOI:** 10.3390/s19194335

**Published:** 2019-10-08

**Authors:** Stefano Laureti, Hamed Malekmohammadi, Muhammad Khalid Rizwan, Pietro Burrascano, Stefano Sfarra, Miranda Mostacci, Marco Ricci

**Affiliations:** 1 Department of Informatics, Modeling, Electronics and Systems Engineering, University of Calabria, Via P.Bucci, Arcavacata, 87036 Rende (CS), Italy; 2 Department of Engineering, Polo Scientifico Didattico di Terni, University of Perugia, 05100 Terni (TR), Italy; hamed.malekmohammadi@unipg.it (H.M.); muhammadkhalid.rizwan@unipg.it (M.K.R.); pietro.burrascano@unipg.it (P.B.); 3 Department of Industrial and Information Engineering and Economics, University of L’Aquila, 67100 L’Aquila (AQ), Italy; stefano.sfarra@univaq.it; 4 Restorer, Via Muranuove 64, 67043 Celano (AQ), Italy; miranda.mostacci.90@gmail.com

**Keywords:** pulse-compression thermography, hyperspectral imaging, defects, cultural heritage, image processing, information fusion, painting on canvas, NDT, principal component analysis, independent component analysis

## Abstract

The use of different spectral bands in the inspection of artworks is highly recommended to identify the maximum number of defects/anomalies (i.e., the targets), whose presence ought to be known before any possible restoration action. Although an artwork cannot be considered as a composite material in which the zero-defect theory is usually followed by scientists, it is possible to state that the preservation of a multi-layered structure fabricated by the artist’s hands is based on a methodological analysis, where the use of non-destructive testing methods is highly desirable. In this paper, the infrared thermography and hyperspectral imaging methods were applied to identify both fabricated and non-fabricated targets in a canvas painting mocking up the famous character “Venus” by Botticelli. The pulse-compression thermography technique was used to retrieve info about the inner structure of the sample and low power light-emitting diode (LED) chips, whose emission was modulated via a pseudo-noise sequence, were exploited as the heat source for minimizing the heat radiated on the sample surface. Hyper-spectral imaging was employed to detect surface and subsurface features such as pentimenti and facial contours. The results demonstrate how the application of statistical algorithms (i.e., principal component and independent component analyses) maximized the number of targets retrieved during the post-acquisition steps for both the employed techniques. Finally, the best results obtained by both techniques and post-processing methods were fused together, resulting in a clear targets map, in which both the surface, subsurface and deeper information are all shown at a glance.

## 1. Introduction

The art of restoration is the final step of a serious conservation approach that should be based on two pillars: the knowledge of art history and the diagnostic of the work of art. Sometimes, the latter is not taken into account by restorers during their work as it is considered expensive and the obtained results are not easy to be interpreted for non-experts in the Nondestructive Testing (NDT) field. However, a constructive and easy interaction between restorers and NDT operators is desired, as it would be beneficial both for the restoration and conservation of cultural heritage goods.

In order to favour the aforementioned interaction, a key role would be played by an exhaustive and “easy-to-be-interpreted” report provided by the scientists to the restorers. This would be highly beneficial, for instance, in interpreting the data coming from ultrasonic testing (UT), wherein echograms are usually preferred to visual images [1,2], or from Ground penetrating (or probing) radar (GPR), which is another NDT method that uses transmitter and receiver antennas for imaging subsurface features. Since the electromagnetic radiation in the microwave band of the radio spectrum is the basis of GPR operation [3,4,5], its use in the cultural heritage field falls into the inspection of thick objects (e.g., ancient floors, mural paintings, historical bridges, etc.). Unfortunately, the interpretation of radargrams is generally non-intuitive to the novice. However, a two-dimensional (2-D) output in the form of an image is usually preferred and desired, as restorers and non-scientific experts may find “defect” positions via the beneficial overlapping of information coming from image fusion [6]. Please note that here the term “defect” may be easily replaced with “covered target”.

Thanks to their thin nature, paintings on canvas allow different types of approaches and methods to be employed. Reflection and transmission modes are both suitable to unveiling information not detectable to the naked eye [7,8]. When working with NDT techniques relying on the use of infrared (IR) radiation, precious quantitative and qualitative information concerning the pictorial, preparatory, and support layers may be retrieved [9].

From the authors’ point-of-view, information coming from the use of active thermography (AT) in the middle-wave IR (MWIR) and hyper-spectral imaging (HSI) in the near IR (NIR) can be fused together in a constructive way. AT is suitable to inspect the whole 3-D structure by analyzing the painting thermal response recorded as time elapses via a thermal camera [10], allowing the detection of splittings, cracks and voids within the multi-layer structure. HSI is able to provide information concerning both the composition of the painting layer in terms of pigments, bindings, etc., and the nature of the preparatory drawing. This is done by exploiting the partial transparency of most of the pigments into the SWIR/NIR [11,12].

The usual question posed by restorers concerning the use of high-power flash lamps (pulsed-thermography—PT) to ensure the necessary signal-to-noise ratio (SNR) is related to the temperature increment (Δ*T*) of the surface after the end of the heating phase, and to the speed of temperature variation. This is because it is important to avoid any thermal stress onto the painting surface and, therefore, the occurrence of possible thermochromism effects. With the aim to avoid those problems, the use of coded excitations to modulate the emission of a low-power light emitting diode (LED) chips system (110 W), in combination with the pulse-compression technique for painting inspection to gently spread the heat stimulus over time was introduced in 2018 by Laureti et al. [13]. This technique is referred as pulse-compression thermography (PuCT), and it was proven to be capable of assuring the inspection capability of PT, while not reducing (and perhaps increasing) the final SNR [14].

With the aim to merge the unique yet complementary capabilities of both the above-mentioned techniques, an integrated use of PuCT and HSI techniques is applied in the present manuscript for the inspection of a painting on canvas sample embedding “covered targets”, such as splittings, cracks, and the so-called pentimenti.

Three novelties are presented here: (i) the use of principal component analysis (PCA) [15,16,17,18,19,20,21] and (ii) independent component analysis (ICA) [22] applied to PuCT output, and (iii) the image fusion of results from (i–ii) and HSI. Points (i) and (ii) may be considered as a further contribution to the scientific knowledge with respect to the works published in [13,23], respectively.

Results obtained via PuCT in the MWIR show clear evidence of fabricated defects at different depths, whilst pentimenti are visible in HSI data working around 1480 nm. As shown in the following, PCA and ICA are implemented directly on the combined PuCT–HSI data to further optimize the information extraction process and, therefore, provide fundamental help to the restorers interested in the combined approach.

The manuscript is organized as follows: the sample object of the study is described in-depth in Section 2; Section 3 and Section 4 provide the main information concerning HSI and PuCT, with details on both the employed setups; in Section 5, the integration between PuCT–HSI and PCA–ICA is given; then, the main obtained results are discussed. Finally, a conclusions section summarizes the main novelties brought to the light for the scientific community.

## 2. Sample Description

**Figure 1:**
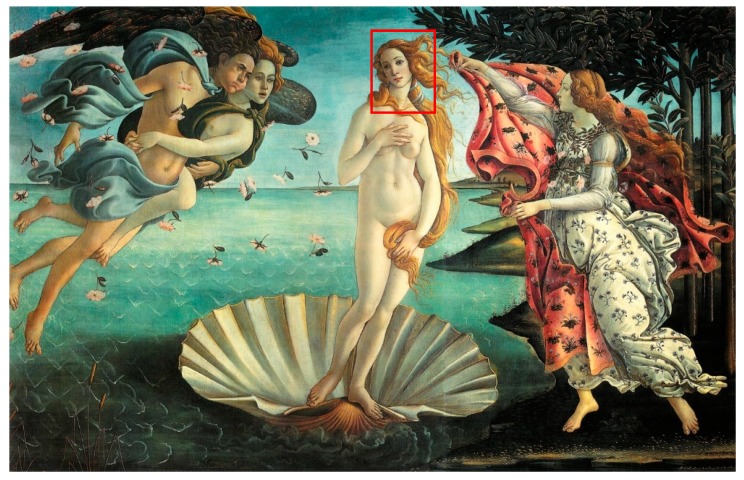
Birth of Venus by Sandro Botticelli. The face of Venus—the red-colored rectangle—is the detail of the painting chosen for the representation in the sample.

For the realization of the sample, the authors chosen a detail of a famous painting by Sandro Botticelli, named “The Birth of Venus” (Figure 1) [24].

The painting is currently preserved at the Uffizi Gallery in Florence (Italy), and dated back to 1482–1485. It was commissioned by the Medici family for the Villa di Castello. It is recognized as being one of the first examples of painting on canvas in fifteenth-century Florence. During the construction of this work, Botticelli preferred a support in linen cloth instead of the typical support consisting of a wooden panel, thus creating a preparation based on plaster on which he executed a tempera painting.

The mock-up investigated here is a pictorial surface realized on a textile support; it was prepared according to techniques of execution in force of an intermediate period in which the passage from painting on wooden supports and textile supports occurred. This era still involves the use of techniques for painting on wooden supports made by gypsum and animal glue, on which a decorative layer realized by tempera is performed. In particular, the tempera layer can be:

- lean: in case glues (animal or vegetal forms) or eggs are used as binders of the pigments.
- fat: in case an emulsion based on egg and oils or resins is used as a binder.

The reason for realising the test sample by employing the methodology described above was to cover a quite extended transition period, so as to make the obtained results potentially valid for different paintings where a combination of painting techniques has been employed.

**Figure 2:**
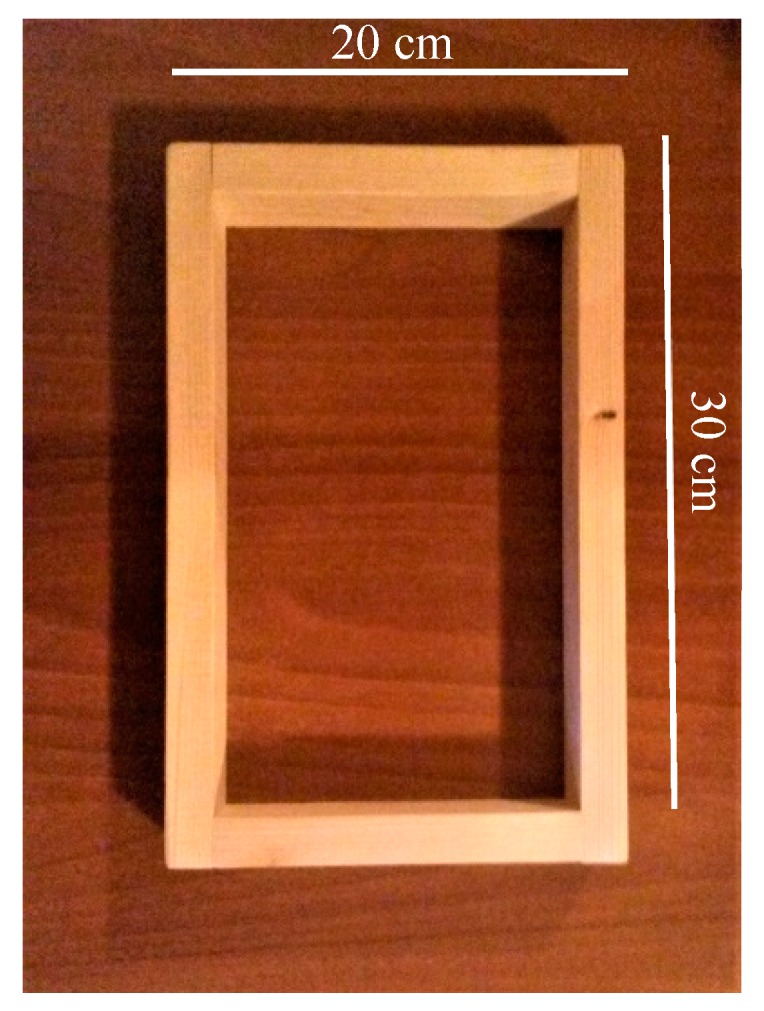
A picture of the frame used for tensioning the canvas.

**Figure 3:**
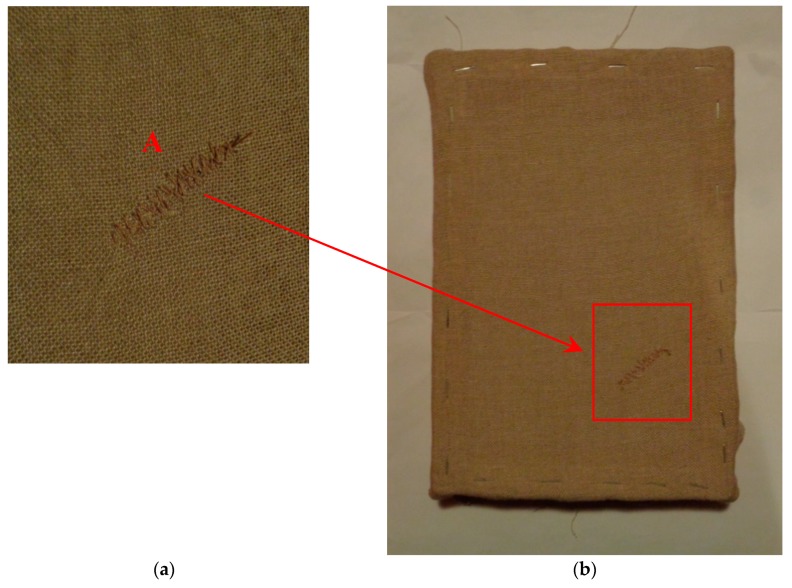
(**a**) Defect A, and (**b**) canvas stretched on the wooden frame.

**Figure 4:**
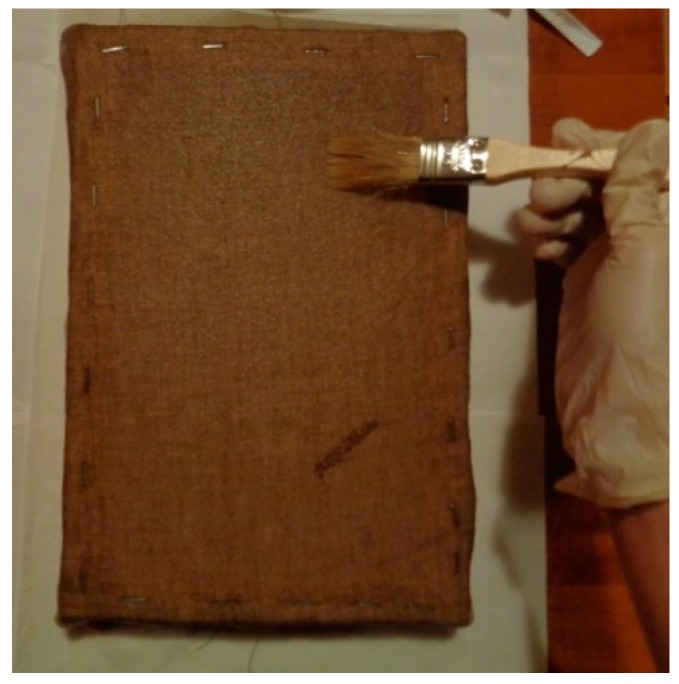
Dressing of the canvas.

**Figure 5:**
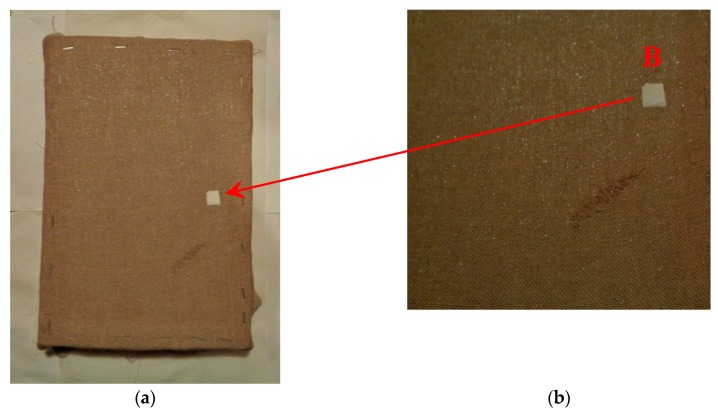
Defect B: (**a**) The defect was obtained by inserting a Teflon insert to simulate a splitting between the preparatory layers; (**b**) a zoom of Defect B.

**Figure 6:**
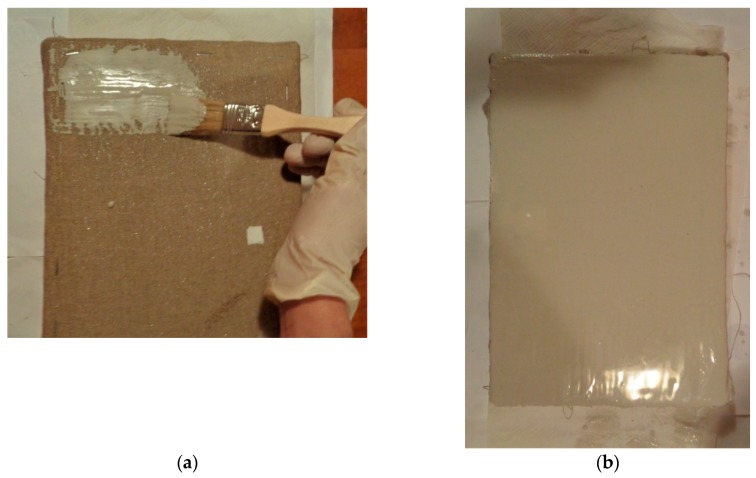
(**a**) Initial step of the application of the first preparatory layer, and (**b**) final step of the application of the first preparatory layer.

**Figure 7:**
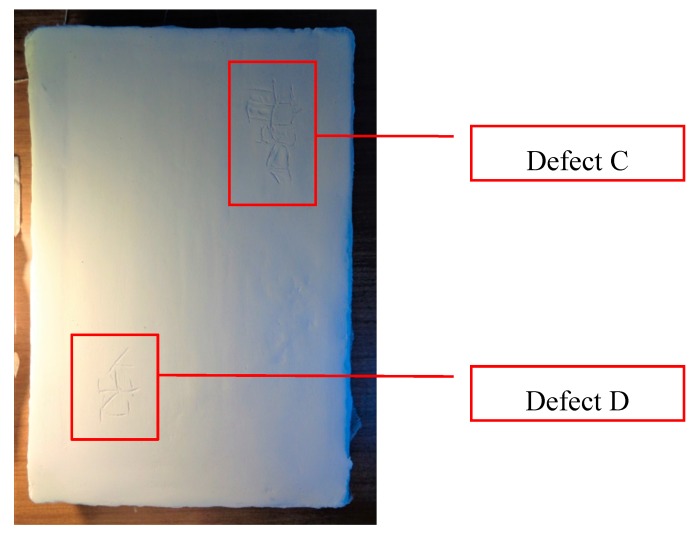
Defects C and D: two dry-crackings were simulated on the still fresh surface.

**Figure 8:**
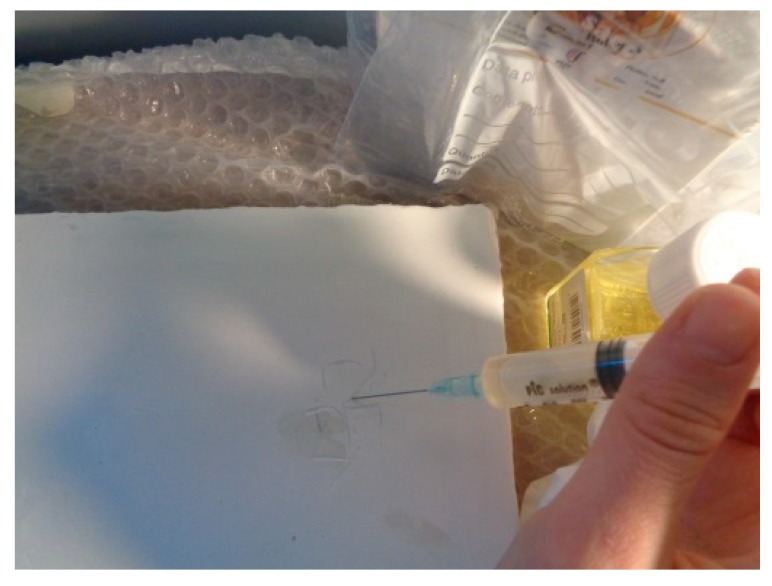
Injection of rabbit glue in the craquelure (Defects C and D).

**Figure 9:**
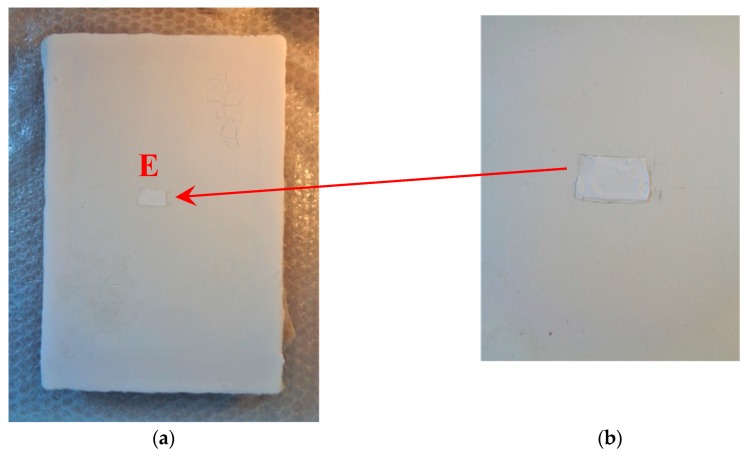
Defect E: (**a**) The defect was obtained by inserting a Teflon sheet to simulate a splitting; (**b**) a zoom of Defect E.

**Figure 10:**
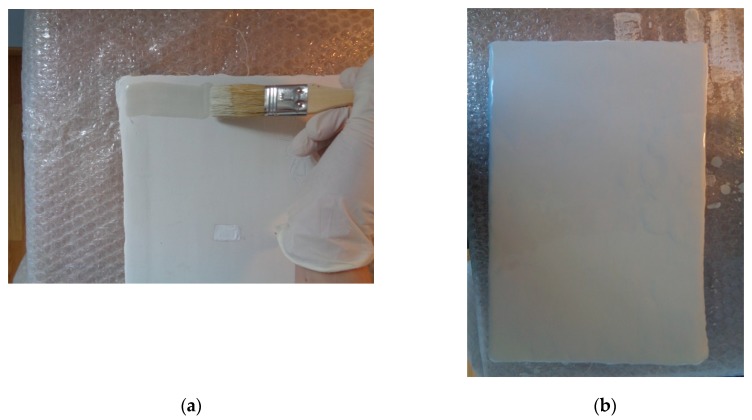
(**a**) Initial step of the application of the second preparatory layer, and (**b**) final step of the application of the second preparatory layer.

**Figure 11:**
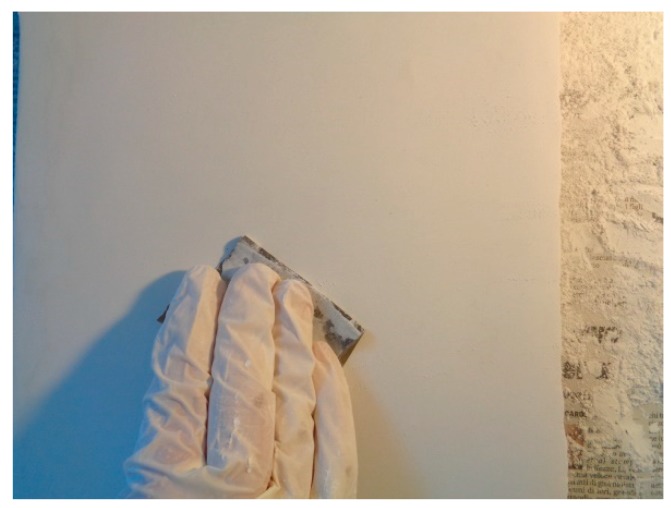
Sanding the surface by means of a fine-grained abrasive paper.

**Figure 12:**
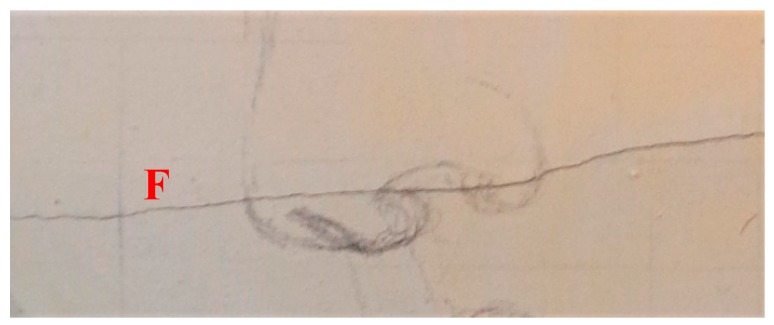
Defect F: crack formed near to Defect E.

**Figure 13:**
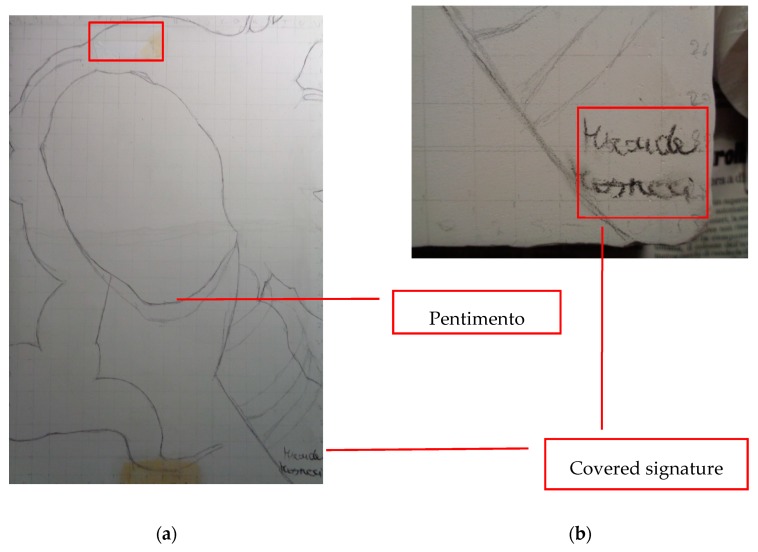
(**a**) Underdrawings and pentimenti, and (**b**) magnification of the covered signature.

**Figure 14:**
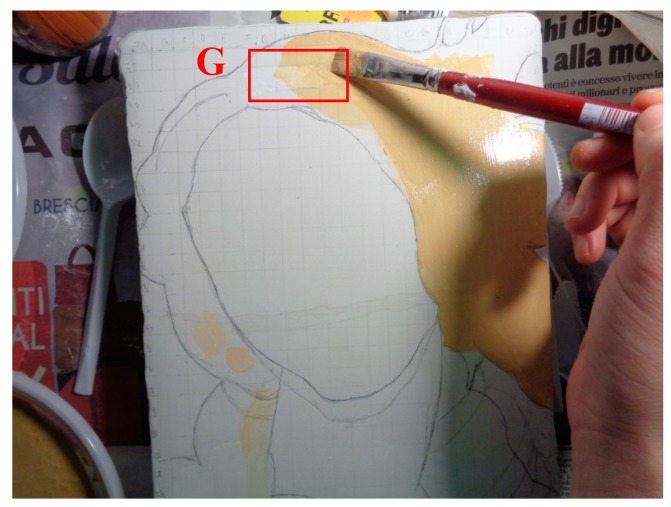
Coating of the primer.

The mock-up sample was fabricated as per the following 20 steps, which are described in detail here below:

(a) The realization of the frame: a wooden frame (20 cm × 30 cm × 2 cm), as shown in Figure 2, was selected and the canvas was tensioned onto it.
(b) Preliminary preparation of the canvas: a rough linen was chosen, and it was cut larger than the wooden frame, frayed along the edges, washed in hot water, dried and ironed. The support was about 1 mm thick and had a regular weft-warp weave in a 1:1 ratio.
(c) Realization of Defect A: as first defect, a diagonal cut of 3 cm long was produced in the canvas and, subsequently, it was stitched to simulate the repair of a torn canvas (Figure 3a).
(d) Tensioning of the canvas: the canvas was tensioned on the frame by means of metal clips applied with a staple gun, fixing one side at a time. Firstly, one side was fixed with a central paper clip, then the same process has been repeated for the opposite side and, finally, this was done also for the remaining two sides; all the sides were pinned using twenty-one staples. Note that the canvas was tensioned with care as the remaining steps influence the final tension itself (Figure 3b).
(e) Dressing of the canvas: since the rough linen does not appear as a surface ready to receive the pictorial layer, a preparation step was required for this purpose. The first step followed the ancient technique, i.e., the canvas was waterproofed with a laying of rabbit glue dissolved in water at a ratio of 1:7 (Figure 4). The glue was left swelling for an entire night in cold water and then heated in a bain-marie to get it completely dissolved. The application of hot glue onto the canvas’s surfaces was carried out using a soft bristle brush. The so-obtained layer was left to dry for 48 h.
(f) Inclusion of Defect B: a Teflon insert with dimensions equal to 1.1 cm × 1.5 cm was placed onto the dried rabbit glue layer and folded up three times, so as to simulate a detachment between the canvas and the next layer of glue and plaster (Figure 5a,b). Defect B is located at about 2 mm depth from the final upper layer, i.e., the inspected surface.
(g) Spreading of the first preparation layer: a layer of about 1 mm of thickness made of Bologna plaster mixed with rabbit glue was applied (Figure 6a,b). The plaster was added to the glue until saturation, i.e., once the desired density was obtained. The laying was done using a soft bristle brush. This layer was left to dry for 48 h.
(h) Realization of Defect C: a network of cracks was crafted onto the previously realized layer with a maximum depth of about 1 mm. This was realized on the fresh plaster layer by means of engravings produced using small plastic chisels (Figure 7).
(i) Realization of Defect D: as for Defect C, a second net of cracks was fabricated on the still fresh plaster layer, this time within a different area of the mock-up (Figure 7).
(j) Sanding of the surface: after a complete drying, the surface was smoothed with a fine-grained abrasive paper.
(k) Isolation of Defect D: an amount of animal glue was injected into the crack network, so as to physically separate the cracks from the next preparation layer (Figure 8).
(l) Insertion of Defect E: as for Defect B, once both the plaster and glue layers were completely dried, a second Teflon insert (dimensions: 1.1 cm × 2.5 cm) was applied on the new surface—it was folded only once on itself (Figure 9a,b).
(m) Spreading of the second preparation layer: above the first preparation layer, a second layer (1 mm thick) of Bologna plaster mixed with rabbit glue was applied in the same way that the first layer was realized (Figure 10a,b), thus completely covering Defect E and all the previously-realized steps.
(n) Sanding of the surface: as for the first layer, the second layer was worked with fine-grained abrasive paper once dried properly; in this way, a surface finishing suitable for receiving the pictorial layer was obtained (Figure 11).
(o) Defect F: a crack with a length of about 15.5 cm was accidentally produced near Defect E; it was deliberately decided to preserve such natural defect and then cover it with the pictorial layer (Figure 12).
(p) Insertion of defect G: the last Teflon insert (dimensions: 1.1 cm × 3.5 cm) was added onto the surface, this time without being folded. It is located between the last preparation layer and the primer for pictorial drawing (see Figure 13a).
(q) Realization of the drawing: once the sample preparation was finalized (i.e., the surface suitable for the pictorial layer was realized), the representation of the selected character was reproduced by drawing the main lines. This was done using charcoal (Figure 13a). As mentioned, the selected detail of Botticelli’s painting is the face of Venus (Figure 1). In addition, a signature of the restorer who fabricated the sample (Figure 13a,b), along with two pentimenti (Figure 13a—see the part around the chin to understand the position of the first pentimento) were added to the sample using charcoal. The second pentimento is in the form of a wrongly positioned eyebrow over the right eye.
(r) Priming: first, a basic colored pattern was obtained for the pictorial layers, which was lighter for the sky and darker for the areas relative to the locks of hair of Venus (Figure 14).

The employed primer was a mixture of powdered pigments and an emulsion of egg yolk and linseed oil. Note that Defect G (Figure 14) was completely covered after the primer was coated.

**Figure 15:**
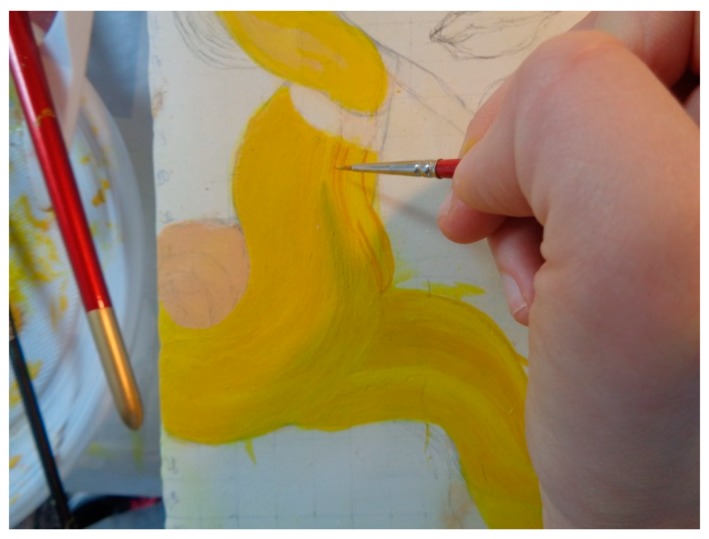
First stage of the application of the painting layer.

(s) Pictorial drawing: for this step, a greasy tempera for painting was used. In addition, powder pigments were added as a binder, together with an emulsion consisting of an egg yolk, a teaspoon of linseed oil and two drops of vinegar. It should be noted that a greasy tempera based on egg and oil was commonly employed in the fifteenth century, especially in a transitional phase from painting on panel to oil painting on canvas. However, it was chosen to add vinegar to guarantee the preservation of the tempera. The mock-up was finally completed by a series of overlapping pictorial backgrounds realized by means of brushes of marten hair (Figure 15), and then by mixing the right amount of pigment diluted in water along with the binder each time.

Inorganic pigments of Winsor and Newton, and Ferrario were used. The colors were obtained from a range of ten pigments: white zinc, lemon cadmium yellow, golden ocher, natural sienna, scarlet red, dark sienna, chrome oxide green, cyan blue, natural umber and ivory black.

**Figure 16:**
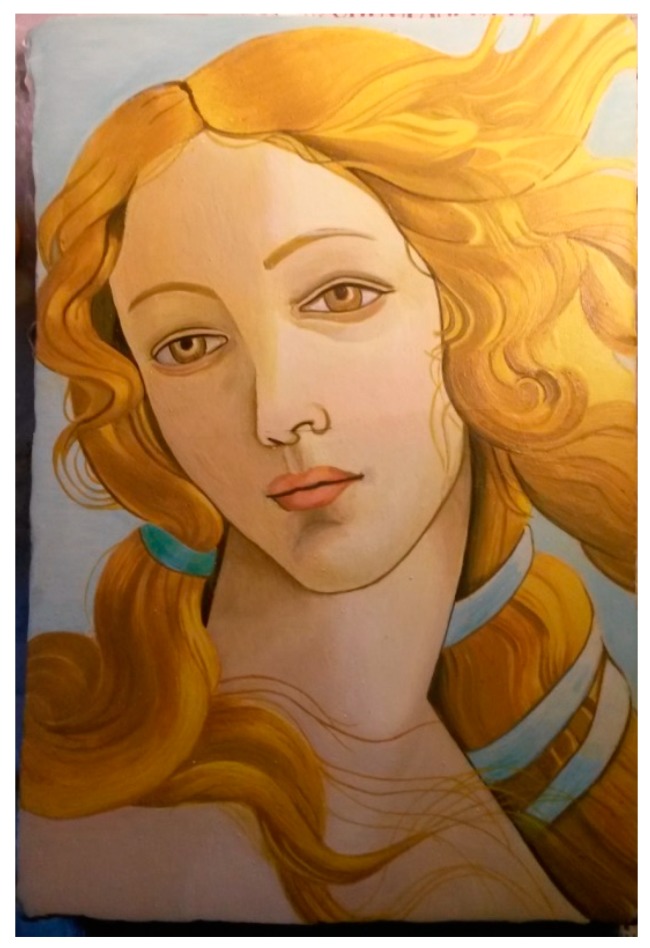
Final sample including the finishing layer.

(t) Finishing: a coat of a natural resin-based paint was spread on the paint layer using a brush (Figure 16).

**Figure 17:**
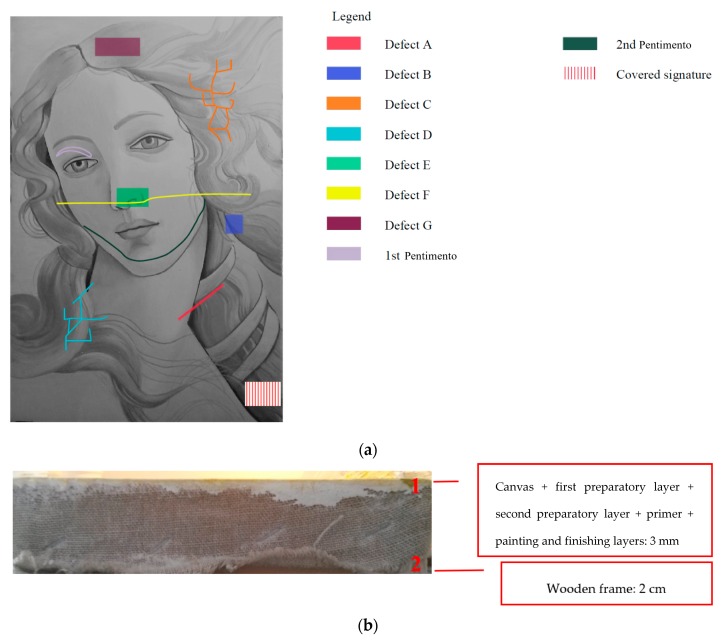
Sample: (**a**) map of the defects/covered targets projected on the horizontal plane, and (**b**) cross-section of the sample describing: (1) the thin layers, and (2) the thick layer.

In this case, a ready-to-be-used product by Maimeri was applied onto the painting layer. To summarize the main information concerning the fabricated defects and thicknesses, the reader can refer to Figure 17a,b. For the sake of simplicity, the first pentimento will be named hereinafter Defect H, the second pentimento will be Defect I, while the covered signature is Defect L (Figure 17a).

In the next section, a brief description of the HSI and PuCT techniques is given. Interested readers may refer to the provided References in order to deepen the main concepts.

## 3. Hyper-Spectral Imaging

A good way to characterize materials, i.e., identify them or define their properties, is to study their interaction with impinging electromagnetic radiation, and in particular the way in which they reflect it back. When the sensor is sensitive over the back-scattered IR spectrum, this process is referred as IR spectroscopy. In IR spectroscopy, the constituent chemical elements of a given target can be differentiated and classified based on their different spectral signatures, which can be seen like “fingerprints” univocally related to a given constituent. Please note that the identification of complex chemical compounds—as in the case of pictorial layers—requires further classification and processing with respect to the analysis of pure chemical elements. This is because the resulting spectral signature shows a more complex spectrum due to the combination of many elements’ “fingerprints”. In this framework, measuring the reflected radiation and performing point-by-point (i.e., pixelwise) Fourier analysis of it is the most common way to extract useful information about the target sample surface and subsurface features. Such analysis can be implemented by means of a hyper-spectral camera that, thanks to the use of a diffraction grating or of a monochromator combined with an IR camera, produces images of a sample in which for each pixel of the image, a discrete spectrum is associated and defined by a certain number of “bins” over the sensor’s sensitivity [25].

It must be noted that most of the HSI systems are designed to perform line-scanning of the sample under test with each frame of the IR camera having N×M pixels, whereby N×1 is the number of pixels that subdivide the imaged part and M is the number of bins in which the sensor spectrum is subdivided. Since cultural heritage objects may have a large surface area, tiling techniques are required to capture the detail of these surfaces [26]. As mentioned previously, HSI cameras usually acquire a single row of pixels at time, and the camera used in this paper does too. Thus, to form various 2-D images of the sample (one for each of the *M* different wavelengths), the sample or the camera must be moved.

**Figure 18:**
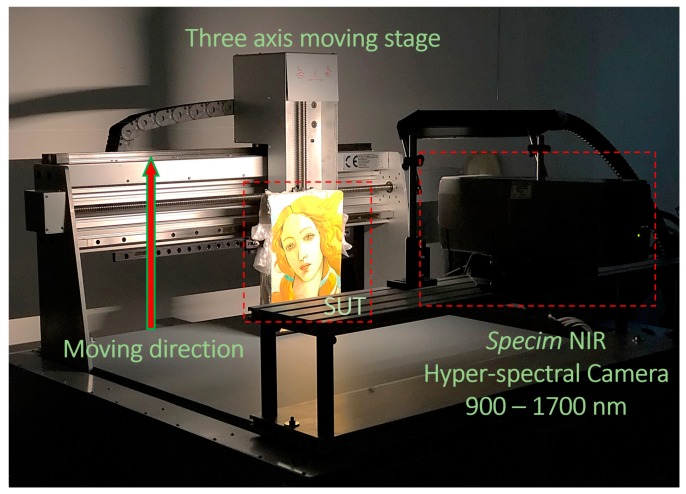
Hyper-spectral imaging experimental setup. SUT = sample under test.

In this work, a Specim NIR hyper-spectral camera (working in the 900–1700 nm spectral range and having 256 spectral bins and an acquisition frame rate of 100 fps) was used along with a commercial 100 W halogen lamp. The light source was placed 2 m from the surface of the sample under test (SUT) and the glass protection was removed to maximize its emission in the NIR range. The SUT was placed on a precision three-axis moving stage (model “Blu8 Jewel” by Delta Macchine Cnc SRL, Rieti, Italy) and moved along his vertical main axis at a constant speed during the acquisition. A labelled picture of the setup is shown in Figure 18. Note that the results shown in Section 6 have been normalized $\left(IM{\left(N,M\right)}_{norm}\right)$ to consider the camera sensitivity, which varies both with pixels and wavelength. Equation (1) shows this process for a single acquired line through the hyperspectral camera IM(N,M):

(1) $$IM{\left(N,M\right)}_{norm}=\frac{IM\left(N,M\right)-IM_{black}\left(N,M\right)}{IM_{white}\left(N,M\right)-IM_{black}\left(N,M\right)},$$

where $IM_{black}\left(N,M\right)$ is a reference spectrum obtained by clogging the camera lens, and $IM_{white}\left(N,M\right)$ is a reference spectrum obtained by illuminating a uniform white reflector placed at the top of the scanned sample. The process described in Equation (1) has been repeated for all acquired lines.

## 4. Pulse-Compression Thermography

Among the most valuable non-destructive evaluation methods for the inspection of cultural heritage (CH) objects, InfraRed Thermography (IRT) currently holds an important place of prominence. Both IR and thermal methods for NDE are based on the principle that the heat flow in a material is perturbed by the presence of anomalies, a good example being detachments. The imaging or the visualization of such thermal imprints is known as IRT [1]. A theoretical definition can be found in the NDE Handbook centered on IRT [27] and adapted as follows: “IRT is a nondestructive, nonintrusive, noncontact technique that allows the mapping of thermal patterns, i.e., thermograms, on the surface of the objects, bodies or systems via an IR imaging instrument, such as a thermal camera”.

IRT popularity has grown in the recent years due to spatial resolution and acquisition rate improvements in thermal cameras; IR cameras are quickly becoming more handy, affordable, and accurate at the same time. Further, by using suitable lenses, IRT allows fast inspections and real-time measurements over either a quite large or a small detection area. At the same time, the development of IRT theory and of advanced image processing techniques focused on the acquired thermograms has enabled the assessment of more and more detailed information in cultural heritage objects [28]. In the active approach, an external heat source is used to stimulate the material being inspected in order to provoke a thermal contrast between defective and non-defective (i.e., the background) areas. This approach is usually adopted in laboratory inspections because the object is assumed to be at thermal equilibrium [29], and routinely high-powered flash head lamps (some kJ’s of energy) are used as the heat source. For non-expert users, there is a high chance to provoke the thermochromism effect on the painting surface, as well as the deposition of too-high thermal stresses, especially using the pulsed thermography scheme [30]. This risk must be avoided or at least highly mitigated because, as an imaging method, IRT allows the conservation analysis [31], the first judgment of constitutive materials and painting technique (whether present) [32], as well as the comparison with additional imaging data [33]. Hence, the challenge is to reduce the power of the heating system without losing the penetration depth and the inspection capability with respect to employing high-powered flash lamps.

In this framework, the use of coded modulated heating stimuli in combination with the pulse-compression technique, also known as PuCT or thermal wave radar imaging [34], has proved to be a beneficial and robust NDT method in which a given heat amount is delivered to the SUT by spreading it over an arbitrarily long time period so as to limit the heat peak power below a certain value [35]. Thanks to the pulse-compression technique, almost the same information of an equivalent pulsed thermography test of the same delivered energy is retrieved and, at the same time, a high flexibility in the heating process is obtained. This feature is extremely attractive when dealing with painting inspection and cultural heritage goods in general, since it allows tuning of the PuCT scheme to cope with the characteristics of the SUT and to avoid any possible thermal stress or thermochromism. To fully exploit this flexibility, a set of LED chips were used as the heat source, as reported in [13,14].

It must be noted that, in both the PuCT and the PT techniques, the heat propagation is usually approximated as a 1-D phenomenon having a negligible contribution to lateral diffusion [36]. Further, concerning the heat transfer in materials related to a pulsed thermal excitation, readers can refer to [37] and [1], respectively.

Taking into account these References, it is possible to understand how an optimal measurement of the impulse response in PuCT (which should be as close as possible to the output of the same test carried out with PT) is not only important for a useful defect detection, but for defect classification purposes too. Indeed, if the excitation pulse is too long, the signal of a single pixel (*j_x_*, *j_y_*) cannot be considered as an impulse response. It should instead be considered as the signal inherent to the convolution of the impulse response with the excitation waveform. This incorrect procedure makes interpretation of the data more difficult, even if it increases the SNR for some defects [38].

**Figure 19:**
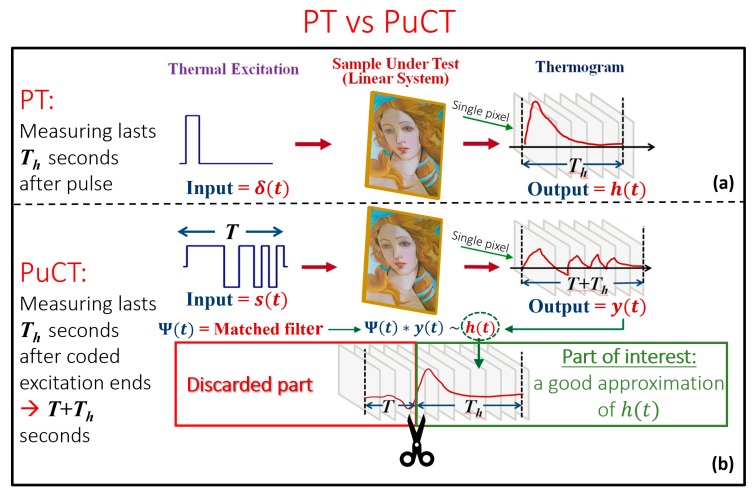
Comparison between (**a**) pulsed thermography (PT), and (**b**) pulse-compression thermography (PuCT).

For the sake of clarity, a graphic comparison between PT and PuCT is reported in Figure 19.

Figure 19a shows that in PT, the excitation is considered instantaneous and the sample impulse thermal response (*h*(*t*)) is measured for time *T_h_*, which is the expected duration of the impulse response of interest, i.e., the time necessary for the diffusion of the heat.

Instead, in PuCT (Figure 19b), the sample is excited with a coded excitation of duration *T*, while the thermograms are collected for an overall time of *T* + *T_h_* [39,40,41,42]. After the application of the PuC algorithm, an estimated impulse thermal response of duration *T_h_* is retrieved.

The PuC technique relies on the assumption that the SUT can be considered as a linear time-invariant (LTI) system. In the mentioned case, the PuC output is an estimate of the impulse response *h*(*t*), but with the advantage to be retrieved even in a noisy environment, or in the presence of very low power peak values. In Figure 19b, a coded excitation s(t) is provided along with another signal (t) (i.e., the matched filter); their convolution (denoted by “*”) approximates the Dirac’s delta function δ(t). The impulse response h(t) estimate, $\tilde{h}\left(t\right)$, is retrieved by exciting the LTI system via the s(t) signal as a first step, and by convolving the system output y(t)—the recorded series of thermograms—with Ψ(t) as a second step. This is mathematically shown in Equation (2), where *e*(*t*) is the additive white Gaussian noise contribution:

(2) $$\tilde{h}\left(t\right)=y\left(t\right)*\Psi \left(t\right)=h\left(t\right)*\underset{=\tilde{\delta }\left(t\right)}{\underset{︸}{s\left(t\right)*\Psi \left(t\right)}}+\underset{=\tilde{e}\left(t\right)}{\underset{︸}{e\left(t\right)*\Psi \left(t\right)}}=h\left(t\right)*\tilde{\delta }\left(t\right)+\tilde{e}\left(t\right)\approx h\left(t\right)+\tilde{e}\left(t\right).$$

**Figure 20:**
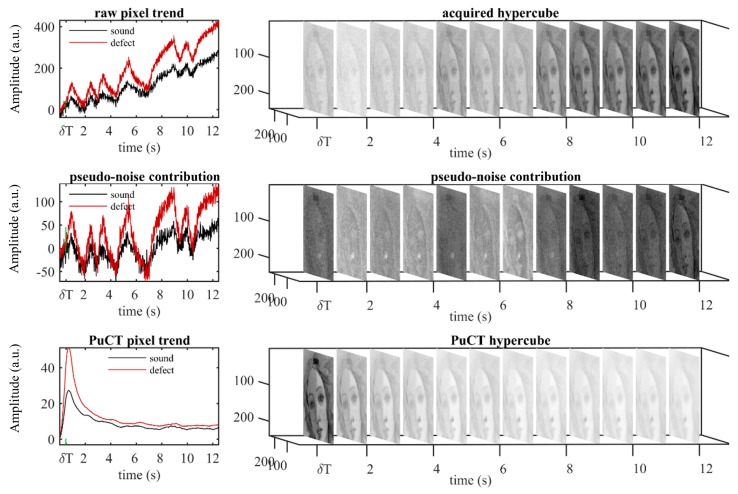
Pseudo-noise pulse-compression thermography (PuCT). Top: hyper-raw thermograms for both a defected and sound pixel. Middle: the same signal as for Top, but after the de-trend procedure, thus ready for the pulse-compression step. Bottom: signals obtained after PuCT. From the series of thermograms showed as time lapses, it is possible to note how the signal-to-noise ratio (SNR) is enhanced from the raw acquired signal to the PuC output.

Concerning the maximization of the achievable SNR, the best choice for the matched filter Ψ(t) is simply given by the expression Ψ(t)=s(−t) [43]. It is now important to underline the main steps to be followed to correctly implement the PuCT procedure, which are represented in Figure 20. Firstly, the sample should be excited with a coded excitation modulated heating stimulus of duration *T*. The coded excitation *s*(*t*) chosen here is a pseudo-noise binary code [44,45,46,47,48,49]. Secondly, thermograms are collected for a time *T* + *T_h_*, where *T_h_* is the expected duration of the impulse response of interest (i.e., the duration of the equivalent PT analysis). Thirdly, taking into account that unipolar heat sources are exploited in the form of low power LED chips, a step heating response contribution must be suppressed in the recorded thermograms before the application of Ψ(t). An efficient fitting procedure based on non-linear polynomial fitting can be implemented to remove this undesired term [39]. The result of the mentioned de-trend procedure is shown in the middle plot of Figure 19, wherein an Alternate-Current “AC” recorded sequence (almost unaffected by a Direct-Current “DC” component) is obtained corresponding to the theoretical response of the SUT to a true bipolar pseudo-noise heat excitation. This signal is now ready for the PuC. The PuC algorithm is finally applied by convolving the pseudo-noise contribution with the Ψ(t) pixel-by-pixel. Thus, an estimation of the pixel impulse response with a duration of *T_h_* is finally obtained. It should be noted that the finite duration of the coded heating stimulus leads to an unavoidable additional mathematical error in the form of side-lobes affecting the retrieved impulse response. This error becomes negligible when *T_h_* is significantly shorter than *T*, although this is not typical in the PuCT method. The spreading of the energy at very low thermal frequencies (from tens to hundreds of mHz) is also required in materials having low thermal diffusivity values. This point limits the SNR achievable by PuCT and, in addition, it is in contrast with the design of the optimal code [47]. On the one hand, it should be noted that it is unsuitable to impose to the code to last a few minutes in order to guarantee high Time-Bandwidth (*TB*) product of the waveform - a good *TB* would be >100 - in case the bandwidth is limited to fractions of Hz. — On the other hand, as the SNR gain provided by PuC is proportional to the parameter *TB*, a trade-off between SNR, side-lobe levels and measurement time must be found [39].

**Figure 21:**
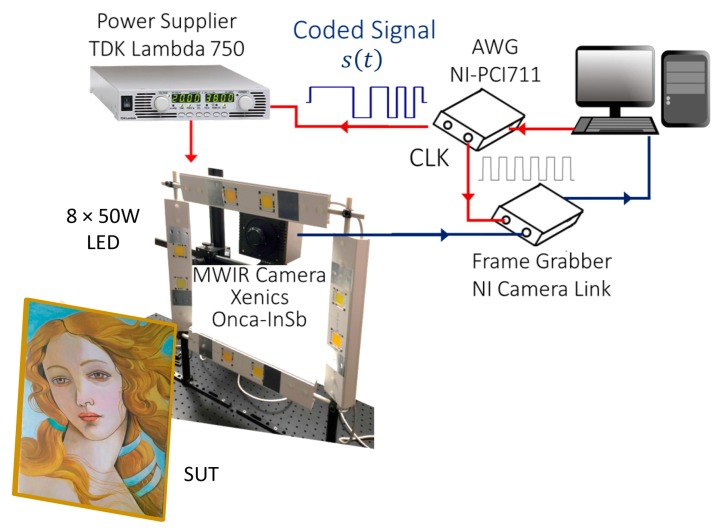
Pulse-compression thermography setup. LED = light-emitting diode.

Concerning the exploited PuCT setup, Figure 21 shows a sketch of the employed equipment:

The signal generation/acquisition was managed by Labview^TM^ software. A Xenics Onca-MWIR (3.6–4.9 μm)-InSb IR 320 × 240 pixels camera was used to record the thermograms. As mentioned previously, eight LED chips were used in reflection mode at a chosen total electrical absorbed power of 110 W. In this way, a low heating rate was assured to the SUT surface. The distance between the painting and the camera was about 50 cm. The main parts of the equipment were synchronously driven by the signals provided by a National Instrument(NI) PCI-6711 Arbitrary Waveform Generation (AWG) board. The AWG was also connected to a NI1433 Camera Link Frame Grabber. Both the AWG board and the grabber were connected to a central PC/Digital Signal Processing unit. The coded excitation was input into a power amplifier consisting of a TDK Lambda GEN 750 W power supply. To understand in-depth how the experimental setup works, readers may refer to [13,39,47].

## 5. Integration Among PuCT, HSI and Post-Processing Analyses

The idea at the base of the integration among PuCT and HSI reflects different motivations. Firstly, it ensures sensitivity to a wide range of painting “defects”; secondly, it allows for exploiting image processing to overcome the limits of each single technique and, lastly, it is useful as a procedure for the in situ inspection in reflection mode. In particular, HSI is strictly related to the pictorial and drawing layers because there is a direct imaging of the IR radiation reflected by the painting (NIR/short-wave IR), while AT in the mid-infrared/long-wave infrared spectrum is linked to preparation, support and intra-layer defects and it is able to image the IR emitted due to thermal excitation too.

The goals, i.e., technical objectives, are as follows: a) developing and exploiting pseudo-noise PuCT to increase SNR while using a low power heating source (LED); b) testing various time-domain processing approaches on thermal data; c) applying multivariate analyses in order to both improve sensitivity and optimally fuse the different information. In fact, a single thermal image is seldom not enough to be shown alone in complex inspection tasks and it is often not self-explanatory; for instance, in scenarios where temperature gradients are small or when dealing with objects that have a high emissivity variation. Thus, additional data such as reflectograms and/or visible and/or hyper-spectral images are required for documentation and in decision-making for artwork restoration/conservation purposes. For instance, visual images can be used for enhancing thermograms since they provide a different representation of the scene (complementary information) [50].

Thermal imaging can also be used as complementary information when the main findings come from another NDT method, and vice versa. Taking this into account, the fusion of thermal and non-thermal information is considered in this work by using the above-mentioned NDT methods. Even by using PuCT, single thermograms obtained by imaging the retrieved impulse responses were not able to show up all the main defects described in Section 2. To increase the defect detection capability, the sequence of the thermograms was further analyzed by applying PCA and ICA, as reported in the literature. For instance, see [15,16,17,18,19,20,21] for the use of PCA in thermography and [51] for the use of ICA, respectively.

In particular, in [51], it is shown how the captured thermal images can be regarded as a set of mixed signals from multiple sources, e.g., materials, defects, uneven heating effects, and noise from different sources. Therefore, there is little dependency among these sources; the signals composing them can be initially separated by ICA and, subsequently, the defect information may be shown in a small number of component images. The influence of inhomogeneous backgrounds and noise can be largely relieved, while the targets are highlighted. ICA was used in the active thermography configuration for analyzing panel paintings [52] and impacted composite materials made by natural fibres [53], both subjected to a preliminary long-pulse radiation. The use of PCA and ICA in combination with PuCT, with both coded or frequency-modulated excitation, was addressed instead by Mulavesaala and co-workers in [54,55], and by some of the present authors in [35].

Compared to other methods, ICA is preferred because it achieves dimensionality reduction, background elimination, and defect feature extraction simultaneously. For the same reasons, ICA and PCA have also been applied to hyper-spectral images and have obtained interesting results, which are shown in the following section.

Please note that the spatial resolution of the IR camera was much lower than that of the hyper-spectral camera. For this reason, the PuCT thermograms were resized with a linear interpolation to match the resolution of the hyper-spectral ones before performing any image fusion.

## 6. Results and Discussion

**Figure 22:**
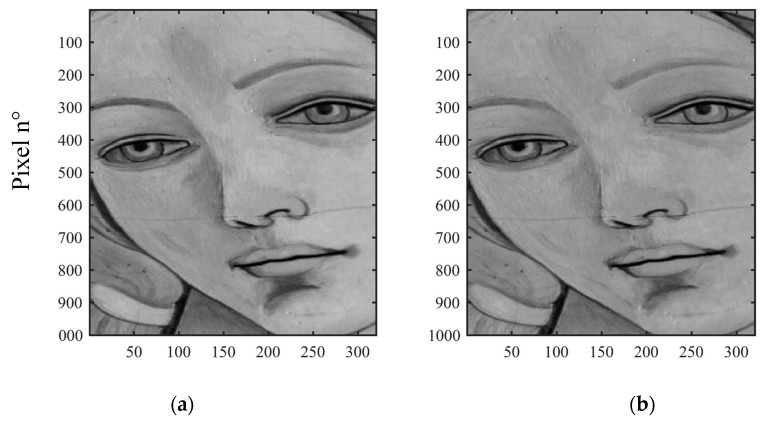

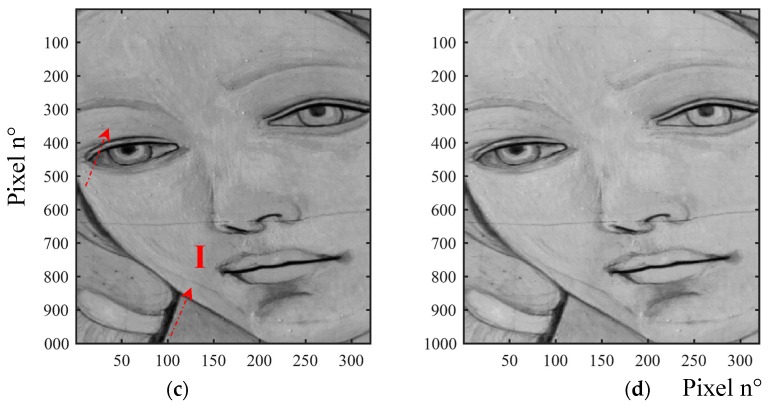
Raw hyper-spectral images acquired at: (**a**) 1100 nm; (**b**) 1200 nm, (**c**) 1400 nm, and (**d**) 1650 nm.

Inspections started with the HSI system. From Figure 22, it is possible to see how the longer the wavelength, the higher the pigment transmission. The drawing and primer layer are more visible; in fact, pentimenti (Defects H and I) both start becoming readable at 1400 nm (Figure 22c) without any post-analysis applied to the captured raw HSI images.

However, pentimenti are more visible at 1650 nm. Although their positions are marked with dotted red arrows, readers may refer to Figure 17a for an overall view.

**Figure 23:**
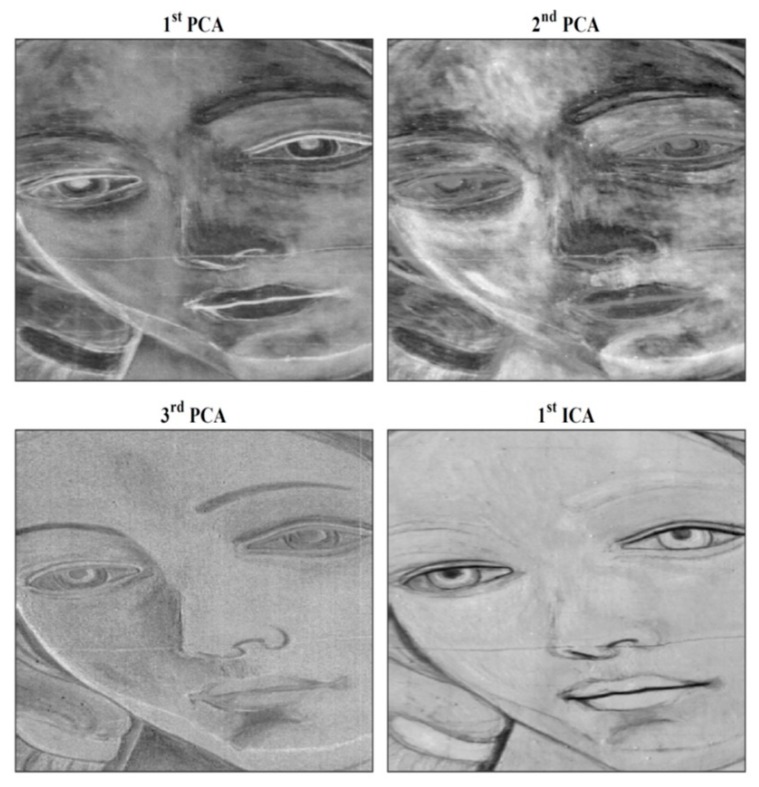
First, second and third PCA applied to hyper-spectral images, and the first ICA applied to the same set (between 1400 nm and 1650 nm).

The defect/hidden target detection can be further improved by applying PC or IC analyses to raw hyper-spectral images. Figure 23 shows the first, second and third PC, along with the first IC. The latter is located at the bottom right hand corner of Figure 23.

By working between 1400 nm and 1650 nm, the results in Figure 23 show the ability to detect different details. The first pentimento is visible in both the first and third PCA, as well as in the first ICA. Pentimento H is also detectable in the first ICA. Crack F appears with great evidence both in the first and third PCA, as well as in the first ICA. The second PCA is able to show the brush strokes of the artist that realised the sample.

**Figure 24:**
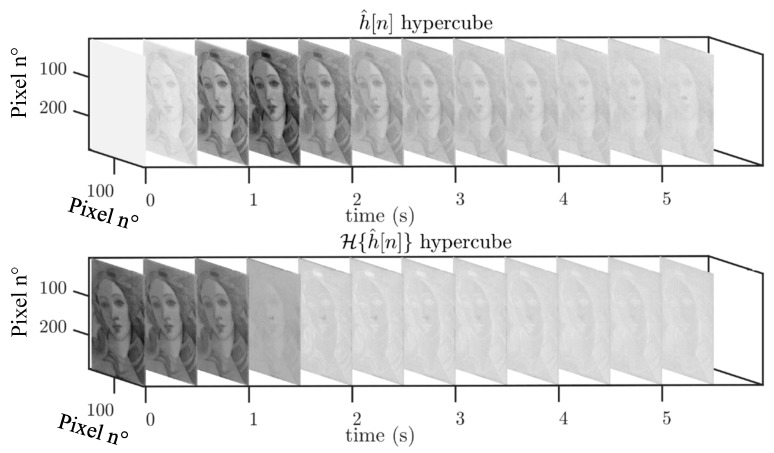
Hypercube showing the *h*(*t*) and the time-phase results as time elapses.

**Figure 25:**
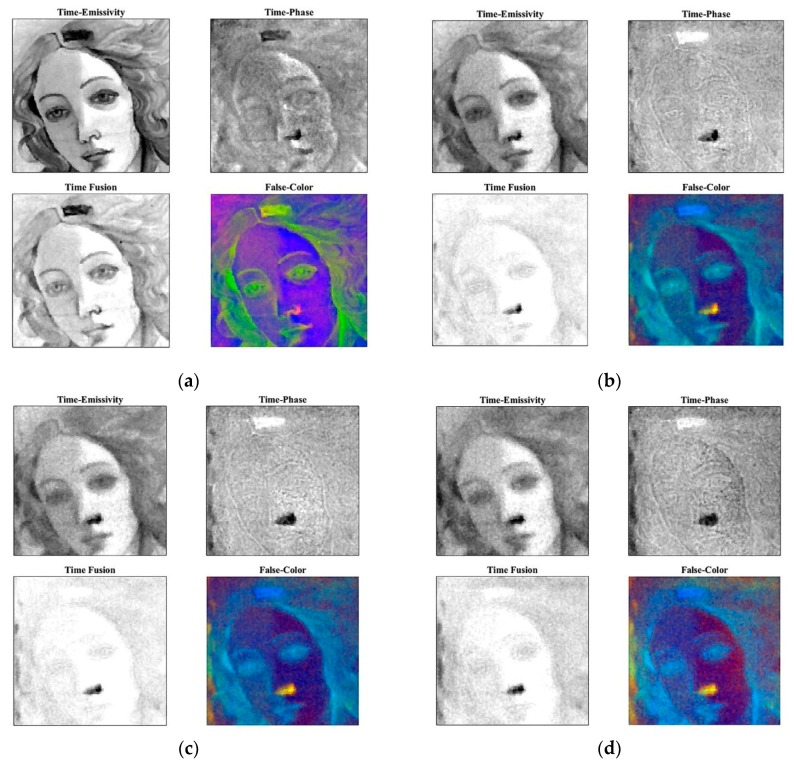
Results extracted from the impulse response H{h(t)} at different times: (**a**) 2 s, (**b**) 22 s, (**c**) 18 s, and (**d**) 26 s.

Concerning PuCT, the most interesting results have been obtained via time fusion by applying the false color technique after having performed the Hilbert transform (H{·}) over the impulse response. This is referred to as a “time-phase” algorithm and is shown in Figure 24 as time elapses. The real (amplitude), imaginary and phase part of the H{h(t)} have been used and displayed as red, green and blue colors, respectively, in Figure 25 (false color). In addition, Figure 25 shows “time-fusion” images at different times. These have been obtained by normalizing each image depicted in “time-emissivity” and “time-phase” subplots to their respective maxima, so as to obtain image levels bounded within a range from 0 to 1. The so-obtained images are then multiplied pixelwise leading to the “time fusion” feature.

Deeper detachments become visible as time elapses from Defect G (shallower) to Defect B (deeper) (Figure 17) and (Figure 25a–d).

Readers may compare the results obtained in Figure 25a–d with the position of the defects shown in Figure 17. The benefit provided by the false-color technique in retrieving the defect positions is evident. In particular, Defects E and G appear easily detectable and evident.

**Figure 26:**
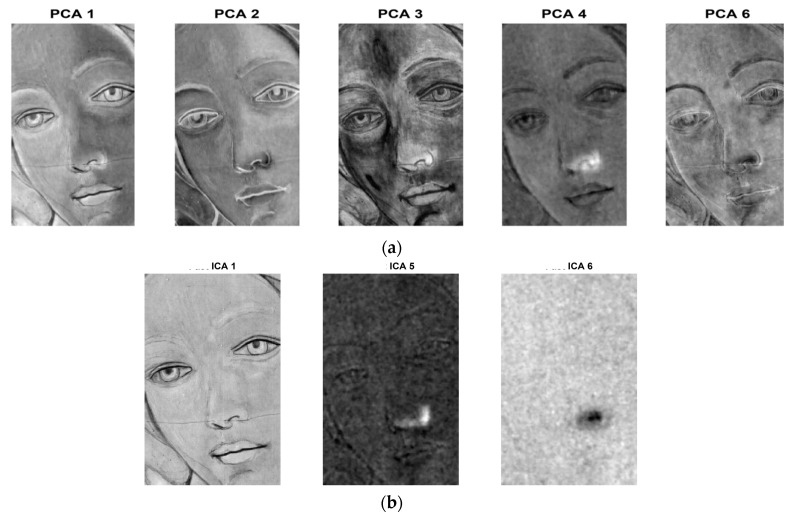
Integration between PC and IC analyses on PuCT images, plus hyper-spectral imaging (HSI).

On the one hand, pentimenti (H and I) cannot be visualized by applying the PuCT method, but also deeper defects cannot be retrieved by means of HSI. Therefore, by integrating the results obtained with the two methods (PuCT and HSI) and, above all, by applying PC (Figure 26a) and IC (Figure 26b) analyses on PuCT images, it is possible to obtain a satisfactory integration between them.

The results have been drawn from a database composed of twelve amplitude images at different times from PuCT output and 140 HSI images from 1200 nm to 1650 nm. Note that the choice of the mentioned set of wavelengths for HSI images was based on the fact that pentimenti appeared clearly within this range (see Figure 22), whilst the selected twelve PuCT images were the same as shown in the bottom part of Figure 24, i.e., thermal images showed the defects the best. The image processing has been focused on a small part of the sample in order to minimize the high computational cost required, but an at-a-glance depiction of most of the defects is achieved.

The remaining parts of the sample will be inspected in the future in order to test additional methods.

## 7. Conclusions

From the results shown in the manuscript, it is possible to say that pseudo-noise pulse-compression thermography allows the impulse response to be reconstructed with high fidelity and with low-power sources, i.e., 100 W power LEDs (see Figure 20). In addition, defects in the inner layers can be detected in reflection mode by means of PuCT, while multivariate analysis improves defect detection by allowing automatic integration of multi-sensor data.

To obtain an exhaustive map of pentimenti and defects, an image fusion between PC and IC analyses on PuCT images plus HSI results was implemented and revealed its good potentialities.

Since the at-a-glance display is very important for restorers to make decisions on the restoration process, time fusion via false color technique has shown promise in this direction; in particular, real, imaginary parts and phase of the Hilbert-transformed impulse responses have been used as red, green and blue channels, respectively. Finally, a fusion of HSI and PuCT was presented for the first time, applying also ICA and PCA analysis.

However, further optimizations are possible. Among these, it is possible to cite: (a) handy-setup for PuCT thermography; (b) source optimization (e.g., VIS plus NIR/SWIR LED)’ (c) better integration (alignment and processing); and (d) full automatic data fusion, analysis and defect detection (through integration of visible pictures. The latter will be the prospect of future works.
